# Dietary Berries and Ellagic Acid Prevent Oxidative DNA Damage and Modulate Expression of DNA Repair Genes

**DOI:** 10.3390/ijms9030327

**Published:** 2008-03-12

**Authors:** Harini S. Aiyer, Manicka V. Vadhanam, Radka Stoyanova, Gerard D. Caprio, Margie L. Clapper, Ramesh C. Gupta

**Affiliations:** 1 James Graham Brown Cancer Center, University of Louisville, Louisville, KY 40202, USA; 2 Department of Pharmacology & Toxicology, University of Louisville, Louisville, KY 40202, USA; 3 Division of Population Sciences, Fox Chase Cancer Center, Philadelphia, PA 19111, USA

**Keywords:** 4-Hydroxy estradiol, DNA damage and repair, dietary intervention, edible berries, raspberries, ellagic acid, polyphenols, ^32^P-postlabeling

## Abstract

DNA damage is a pre-requisite for the initiation of cancer and agents that reduce this damage are useful in cancer prevention. In this study, we evaluated the ability of whole berries and berry phytochemical, ellagic acid to reduce endogenous oxidative DNA damage. Ellagic acid was selected based on >95% inhibition of 8-oxodeoxyguosine (8-oxodG) and other unidentified oxidative DNA adducts induced by 4-hydroxy-17ß-estradiol and CuCl_2_ in vitro. Inhibition of the latter occurred at lower concentrations (10 μM) than that for 8-oxodG (100 μM). In the in vivo study, female CD-1 mice (n=6) were fed either a control diet or diet supplemented with ellagic acid (400 ppm) and dehydrated berries (5% w/w) with varying ellagic acid contents – blueberry (low), strawberry (medium) and red raspberry (high), for 3 weeks. Blueberry and strawberry diets showed moderate reductions in endogenous DNA adducts (25%). However, both red raspberry and ellagic acid diets showed a significant reduction of 59% (p < 0.001) and 48% (p < 0.01), respectively. Both diets also resulted in a 3–8 fold over-expression of genes involved in DNA repair such as xeroderma pigmentosum group A complementing protein (XPA), DNA excision repair protein (ERCC5) and DNA ligase III (DNL3). These results suggest that red raspberry and ellagic acid reduce endogenous oxidative DNA damage by mechanisms which may involve increase in DNA repair.

## 1. Introduction

Estrogens are involved in the development of several cancers, especially those of the female reproductive system [1]. Breast, uterine and ovarian cancers cause a combined mortality of over 62,000 women in the United States [1]. Apart from its growth stimulatory effects, estrogen is also implicated in other etiological aspects of cancer [2]. It has been shown that 17ß-estardiol (E_2_) and its metabolites can lead to mutations by increasing the rate of DNA damage [3] as well as decreasing DNA repair [4]. Metabolites of E_2_ such as 2- and 4-hydroxy estradiol (4E_2_), can cause oxidative DNA damage in the presence of Cu^2+^ *in vitro* [5, 6]. Further, these metabolites have been detected *in vivo* during mammary tumorigenesis [7, 8]. Thus, they may be linked with the development of estrogen-mediated carcinogenesis. Since DNA damage is a key step in the initiation of cancer, effective inhibition of this damage may be a useful prevention strategy.

There are several methods available to assess DNA damage. Among these, ones that combine a chromatographic method with mass spectrometry have been used to measure numerous products simultaneously [9]. Also, ^32^P-postlabeling in conjunction with polyethyleneimine (PEI)-cellulose thin-layer chromatography (TLC), can be used to measure oxidative DNA damage of various DNA bases, including the benchmark oxidative lesion 8-oxo-2′-deoxyguonosine (8-oxodG) [10–12]. Recently, we have discovered several novel polar DNA adducts by ^32^P-postlabeling and low-salt PEI-cellulose TLC [12, 13]. Although these adducts are as yet unidentified, chromatographic comparison with oxidative DNA adducts formed by Fenton-type reaction (Cu^2+^-H_2_O_2_) suggest that some of the polar tissue adducts may be oxidative adducts [12, 13]. These adducts can be used as a biomarker for selection of antioxidants. Earlier studies from this laboratory have successfully used detection of DNA damage in conjunction with a cell-free system to rapidly screen for chemopreventive/antioxidant agents, thus expediting the process of agent selection. We have been successful in using a tiered approach in reducing benzo[*a*]pyrene-induced DNA damage. Agents such as oltipraz and ellagic acid that were effective in an enzymatic cell-free system were effective in both cell-culture and *in vivo* [14–16].

There are several surrogate biomarkers available to assess the efficacy of dietary agents in a biological system. Among these, the liver due to its proximity and role in the first-pass mechanism represents a suitable surrogate tissue. In addition, due to its high metabolic activity, liver is constantly exposed to the oxidative by-products of cell metabolism, thus making it a suitable tissue to assess the modulation of baseline endogenous oxidative DNA damage by dietary agents.

We undertook an exploratory study to assess the efficacy of dietary berries, a rich source of ellagic acid [17] and pure ellagic acid to reduce endogenous DNA damage in a surrogate tissue (liver) of CD-1 mice to determine if these agents could be used as potential chemopreventive agents against estrogen-mediated carcinogenesis. Female CD-1 mice are highly susceptible to catechol estrogen-induced uterine carcinogenesis [18]. Initially, several agents were tested in an *in vitro* system involving induction of oxidative DNA damage by redox cycling of 4E_2_ catalyzed by CuCl_2_. The most efficacious agent ellagic acid (>95% inhibition of oxidative DNA damage) and its natural source (berries) were then employed in a short-term, *in vivo*, dietary intervention study. The modulation of DNA damage both *in vitro* and *in vivo* was assessed by ^32^P-postlabeling/TLC. In addition, the possible mechanisms by which these agents modulate DNA damage *in vivo* were explored by gene-expression analyses using microarray technology.

## 2. Results

### 2.1 Modulation of 4E_2_/CuCl_2_-induced oxidative DNA adducts by ellagic acid

**Figure 1.**
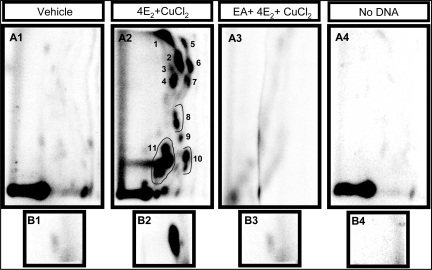
**Representative 32P-labeled DNA adduct maps of both uncharacterized oxidative adducts (A1-A3) and 8-oxodG (B1–B3) generated by redox cycling of 4E2 and CuCl2 in vitro.** The unidentified adducts (5 μg DNA) and 8-oxodG (0.5 μg) were ^32^P-labeled and separated using two directional PEI-cellulose TLC. D1 (bottom to top) and D2 (left to right) solvents were as described in Materials and Methods.

Analysis of DNA damage, induced by redox cycling of 4E_2_ in the presence of Cu^2+^, revealed several unidentified oxidative adducts and 8-oxodG (Figure 1, A1–A3, B1–B3). These adducts were chromatographically similar to adducts generated by treatment of DNA with H_2_O_2_/CuCl_2_ (unpublished data). Neither 4E_2_ nor Cu^2+^ by themselves showed a significant increase in the baseline adduct levels (data not shown). The level of unidentified polar adducts and 8-oxodG in the untreated *st*-DNA (9.7 ± .03/10^6^ N and 11.5 ± 0.85/10^6^ N, respectively) increased significantly after treatment with 4E_2_ and CuCl_2_ (985 ± 54/10^6^ N and 1349 ± 189/10^6^ N, respectively; p<0.05).

**Figure 2.**
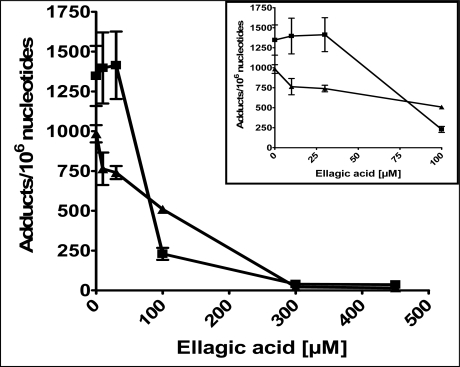
**Effect of increasing concentrations of ellagic acid on oxidative DNA damage.** Both unidentified oxidative adducts (triangles) and 8-oxodG (squares) were measured using ^32^P-postlabeling/TLC and are represented as mean ± SE of 4 replicates. The inset shows the effects at lower concentrations. The test for linear trend (Scheffe’s t-test) was statistically significant with a p-value <0.0001.

Several phytochemicals were tested for their free radical-scavenging potential, initially at a concentration of 300 μM (data not shown), based on earlier studies [16, 19]. In the initial screening, ellagic acid was found to be the most effective, showing >95% reduction of both unidentified oxidative adducts and 8-oxodG compared to the vehicle control (Figure 1A3, B3; Figure 2, p<0.05). This agent also showed a dose-dependant modulation of DNA damage (Figure 2). The unidentified polar adduct levels were diminished by 25% at an ellagic acid concentration of only 10 μM, but significant reductions in 8-oxodG levels were observed only at 100 μM (Figure 2 inset). Based on these results, ellagic acid was selected for a short-term *in vivo* study.

### 2.2 Modulation of baseline endogenous oxidative-DNA damage by ellagic acid and berries

**Figure 3.**
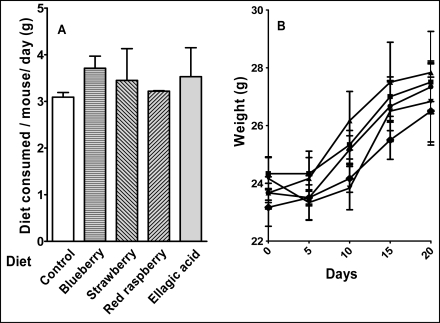
**Comparison of diet intake (A) and weight gain (B).** Female CD-1 mice were fed either control diet (circles) or diets supplemented with 5% (w/w) strawberry (diamonds), blueberry (squares), red raspberry (pyramids) or 400 ppm ellagic acid (triangles). The variance was omitted to enable clear presentation and the differences were statistically insignificant.

**Figure 4.**
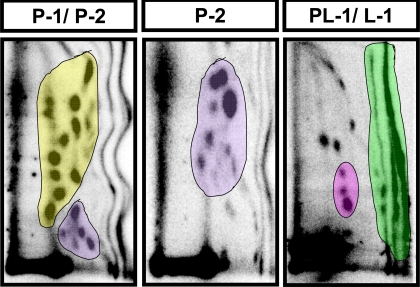
**Representative maps of** **^32^P-labeled endogenous DNA adducts from liver of CD-1 mice.** Mice were fed either a control diet or diet supplemented with strawberry, blueberry or red raspberry (5% w/w each). Labeled adducts were separated by 2-D PEI-cellulose TLC as described in Materials and Methods. Adducts were classified into different subgroups (P-1- yellow; P-2- purple; PL-1- pink; L-1- green) based on their polarities, which was in the following descending order, P-1 >P-2 > PL-1 > L1 as described [13]

There was no significant effect of supplementation with berries or ellagic acid on the diet intake (Figure 3A) or weight gain (Figure 3B), indicating both the absence of toxicity at the doses tested and variation in caloric density of the different diets. All groups represented qualitatively similar adduct pattern (Figure 4). The baseline levels of different subgroups of adducts in the liver of mice fed control diet varied > 20 fold: P-1, 4810 ± 596; P-2, 3398 ± 316; PL-1, 197 ± 32 and L-1, 3074 ± 697 per 10^9^ nucleotides (Table 1). PL-2 adducts were too low to be quantified and 8-oxodG and L-2 adducts were not analyzed. Red raspberry diet significantly reduced all subgroups of adducts analyzed with a 63% reduction of P-1 adducts (p<0.05), 68% reduction of P-2 adducts (p < 0.001), 57% reduction of PL-1 adducts and a 42% reduction of L-1 adducts (Table 1). Ellagic acid showed similar effects albeit at a lower level with 57% reduction in P-1 adducts (p<0.05), 64% reduction in P-2 adducts (p < 0.01), 49% reduction in PL-1 adducts and 17% reduction in L-1 adducts. Blueberry and strawberry diets had moderate but statistically insignificant effects on all adduct subgroups (Table 1).

Four different subgroups of unidentified polar DNA adducts were quantified. The relative adduct labeling (RAL) was calculated for individual adducts in each subgroup as described in Materials and Methods and summed to represent the total adduct level for that subgroup. In case of L-1 adducts the entire region was marked, as indicated in figure 4 and the RAL calculated. Statistically significant results are indicated and the p-value given.

### 2.3 Modulation of gene expression by red raspberry and ellagic acid diets

**Figure 5.**
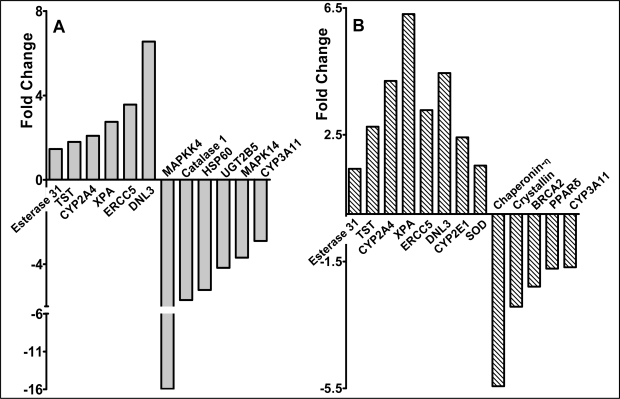
**Hepatic genes with significantly altered expression in female CD-1 mice fed diets supplemented with either 5% (w/w) raspberry (A) or 400 ppm ellagic acid (B) compared to the control diet.** Complimentary DNA synthesized from mRNA was hybridized to Atlas™ Mouse stress array as described in Materials and Methods. TST-Thiosulphate sulphur transferase; XPA- Xeroderma Pigmentosum group A complementing protein; ERCC5 - Excision repair cross complementation group 5; DNL3 - DNA Ligase III; SOD –Superoxide Dismutase, extracellular; MAPK- Mitogen activated protein kinase; MAPKK-MAP Kinase kinase.

Following the similar trend in modulation of adduct levels by both red raspberry and ellagic acid, limited gene expression analysis was performed to determine effect of the intervention on genes involved in DNA repair and xenobiotic metabolism. Microarray analysis revealed that several genes were modulated in a similar fashion by both diets. In particular, genes involved with DNA repair such as – xeroderma pigmentosum group A complementing protein (XPA), DNA ligase III (DNL3), DNA excision repair protein (ERCC5)- were found to be over-expressed by 3 to 8 fold (Figure 5 , p<0.05). There was a significant similarity in the number of genes over- or under-expressed by both diets (Figure 5). In addition, red raspberry diet down-regulated genes such as Mitogen activated protein kinase 14 (MAPK14) and MAP kinase kinase (MAPKK), involved in key cell-signaling pathways, by 5–15 fold (Figure 5).

## 3. Discussion and Conclusions

The purpose of this study was to explore selection of chemopreventive agents against estrogen-mediated carcinogenesis using a semi-tiered approach. First, we used an *in vitro* system involving induction of DNA damage by a carcinogenic estrogen- metabolite (4E_2_) to rapidly screen for a highly effective antioxidant. Then we implemented the results from this tier in a short-term *in vivo* study. The analysis of oxidative DNA damage in both systems provided the mechanistic link to test the biological effects of test agents. CD-1 mice, which are highly susceptible to estrogen-induced carcinogenesis, were used so that the results may be applied to other estrogen-induced malignancies as well. Also, we used a surrogate tissue (liver) instead of a target tissue in order to get an initial assessment of the biological effects of ellagic acid, a compound known to have limited bioavailability [20].

The induction of oxidative DNA damage by 4E_2_ in the presence of Cu^2+^ is postulated to involve hydroxyl radicals [21]. The pattern of polar oxidative adducts generated *in vitro* using either 4E_2_/CuCl_2_ or H_2_O_2_/CuCl_2_ was similar as determined by co-chromatography [11], indicatng that adducts in 4E_2_ and CuCl_2_ reaction originated from oxidative mechanisms. Several studies indicate that H_2_O_2_/Cu^2+^ as well as Cu^+^/Cu^2+^ redox cycling is involved in the generation of reactive oxygen species (ROS) by 4E_2_ [22, 23]. The *in vitro* results from this study correlates well with previous studies from this laboratory in which ellagic acid significantly reduced 8-oxodG induced by H_2_O_2_/CuCl_2_ [19]. The trend in induction of both unidentified oxidative adducts and 8-oxodG were similar, but the relative levels of 8-oxodG was higher in 4E_2_/CuCl_2_ reaction. Copper ions are known to be associated with purine bases in the DNA, thus imparting site specificity for oxidation of guanine bases[23, 24]. Earlier studies from this laboratory have demonstrated that catechols of polychlorinated biphenyls can lead to singlet oxygen-mediated 8-oxodG generation in the presence of Cu^2+^ [24]. DNA bases other than guanine are equally predisposed to oxidative damage [25] and the unidentified polar adducts may be the result of such damage. Using the current technique we were able to quantify total oxidative DNA damage along with selective measurement of benchmark lesion, 8-oxodG.

Ellagic acid, a polyphenol present in berries, is very effective in reduction of 8-oxodG [19]. In this study, it showed a dose-dependant modulation of many oxidative DNA adducts and reduced the levels of unidentified adducts even at 10 μM, whereas a much higher concentration of ellagic acid (100 μM) was required to inhibit 8-oxodG (Figure 2-Inset), suggesting that these 2 types of oxidative lesions develop via separate mechanisms. Singlet oxygen is known to play a predominant role in the generation of 8-oxodG, whereas the hydroxyl radical causes a more promiscuous damage to all DNA bases [21, 22]. Thus, ellagic acid may be more effective in protecting against hydroxyl radical-induced DNA damage at lower concentrations. It is also reported that ellagic acid covalently binds with DNA, but with a higher affinity to poly (dA X dT) than poly (dG X dC) [26, 27]. Such selective interactions with the DNA bases may also explain its differential effects at lower doses. Furthermore, it has already been reported that similar mechanisms are involved in the prevention of carcinogen-DNA adducts by ellagic acid [28], in addition to alteration of carcinogen metabolism [29, 30].

Liver is the primary organ involved in the first-pass mechanism and is affected by both harmful and protective components of the diet. It is also a highly metabolic organ that may possess high levels of endogenous oxidative DNA damage resulting from normal metabolism. Therefore, the ability of any dietary component to reduce the levels of endogenous oxidative DNA damage at baseline would make it an ideal preventive agent in the presence of additional oxidative stress. In this study, we analyzed the levels of at least 4 subgroups (P1, P2, PL-1 and L-1) of novel, unidentified polar DNA adducts (Figure 3). Although these adduct subgroups have not been identified, studies from this laboratory show that all of these adduct subgroups increase during the inflammation of the cervix, suggesting that oxidative stress may play a role in the generation of these adducts [12]. Also, in a separate study, both P-1 and 8-oxodG were induced in the liver of female ACI rats (n=4) after a 12-week treatment with 17ß-estradiol implants (22,000 ± 7600 and 14,000 ± 4400, respectively; p<0.001) compared with sham-treatment (10,000 ± 1200 and 5000 ± 2500, respectively) (H. Aiyer and R. Gupta, unpublished data). Similar trends in the induction of both P-1 and 8-oxodG after E_2_ treatment *in vivo* suggest that they may also originate from oxidative stress. Further, this increase was significantly inhibited by dietary ellagic acid (7800 ± 3300 and 3000 ± 2000, respectively; p<0.001) (unpublished data), suggesting that dietary ellagic acid is highly effective in reducing hepatic DNA damage caused by E_2_ treatment.

To determine if berries and ellagic acid would protect against oxidative stress, we tested their efficacy in reducing endogenous DNA damage in the liver of CD1 mice fed diet containing 5% (w/w) of different berries, or 400 ppm ellagic acid. The berries investigated have different ellagic content – raspberries (1500 ppm ellagic acid), strawberries (500 ppm ellagic acid) and blueberries (<100 ppm ellagic acid)[17, 31]. The dose of ellagic acid was selected based on earlier work by Stoner and colleagues who showed that 400 ppm of dietary ellagic acid, when fed to rats for 23 days, showed significant reduction in hepatic P450 content [32]. Further, the same dose was also effective in reducing N-nitrosomethylbenzylamine (NMBA)-induced esophageal tumors [33]. In this study, both red raspberry and ellagic acid elicited similar effects in reducing the baseline oxidative DNA damage. Since raspberry has the highest ellagic acid content among the berries tested and showed similar effects as pure ellagic acid, we explored the possibility of a shared mechanism in reducing DNA damage. Gene-expression analyses suggested that this effect may partly be due to up-regulation of DNA repair genes (Figure 5). Also, both red raspberry and ellagic acid modulate several genes in a similar fashion, suggesting that the ellagic acid content of raspberries may play a role in their effectiveness. It should however be noted that the concentration of ellagic acid available in the diet through raspberry is 5-fold lower than diet supplemented with pure ellagic acid (75 ppm versus 400 ppm), which shows that ellagic acid present as ellagitannins in berries, may be more bioavailable from berries. This is further confirmed by the moderate effects of both blueberry and strawberry diets on total DNA damage. Indeed 5% strawberries were more effective than 400 ppm ellagic acid in reducing NMBA-induced esophageal tumors suggesting improved bioavailability from a natural source [33, 34] and blueberries, which contain the least ellagic acid were ineffective against the same [35]. Alternatively berries also contain varying levels of anthocyanins that are known antioxidants [31, 36], which may also account for their effectiveness. Ellagic acid is protective against hepatic metal-toxicity and carbon tetrachloride-induced liver fibrosis, where oxidative stress is implicated in the pathogenesis[37–39]. It is clear from our results that ellagic acid and raspberry are hepato-protective via similar mechanisms and are highly effective in reducing baseline endogenous oxidative DNA damage. These effects may, in part, be due to up-regulation of DNA repair genes. Although numerous *in vitro* studies have examined the effects of different polyphenols on DNA damage and repair, conclusive *in vivo* evidence is still lacking [40–42]. Our current study supports the role of berry polyphenols in sustaining genomic stability *in vivo*.

### Conclusion

This study shows that ellagic acid is highly effective in preventing oxidative DNA damage both *in vitro* and *in vivo*. The prevention of oxidative damage induced by 4E_2_, a potentially carcinogenic metabolite in estrogen-mediated cancers, suggests that ellagic acid may be effective in hormonal carcinogenesis. The differential modulation of unidentified oxidative adducts versus 8-oxodG *in vitro*, suggests that ellagic acid is more effective in the inhibition of hydroxyl radical-induced DNA damage than singlet oxygen-induced damage. Dietary ellagic acid is highly effective in reducing baseline hepatocellular oxidative DNA damage partly via enhanced DNA repair. Red raspberry, a natural source of ellagic acid, is more efficacious than pure ellagic acid and also causes upregulation of DNA repair enzymes, making it a suitable candidate for nutritional intervention. Both berries and ellagic acid significantly reduce hepatic oxidative DNA damage, suggesting their usefulness in other hepatic pathologies in which oxidative mechanisms are implicated.

## 4. Materials and Methods

### 4.1 Chemicals

Ellagic acid, dimethyl sulfoxide (DMSO) and salmon testes (*st*-) DNA were purchased from Sigma Chemical Company (St. Louis, MO). 4-Hydroxy estradiol was purchased from Steraloids, Inc. (Newport, RI). Chemicals involved in ^32^P-postlabeling were purchased from sources described earlier [43]. All chemicals used were > 95% pure and were used without further purification. *st*-DNA was freed from contaminating RNA and protein prior to use, as described previously [43].

### 4.2 Induction of DNA damage by 4E_2_/CuCl_2_

*st*-DNA (300 μg/ml) in 10 mM Tris-HCl, pH 7.4 was pre-incubated with vehicle (DMSO) alone and ellagic acid dissolved in DMSO at different concentrations (0–300 μM) and CuCl_2_ (100 μM) for 15 min at 37°C. Redox-cycling was initiated by the addition of 4E_2_ (100 μM) in ethanol. After incubation at 37°C for 1h, DNA was purified by solvent-extraction and ethanol precipitation as described [12, 43].

### 4.3 Animals and diet

Eight week-old female CD-1 mice were purchased from Harlan-Sprague Dawley (Indianapolis, IN). After the initial acclimation period of 1 week, 5 groups (n=6) were fed *ad libitum*, either a control diet or diet supplemented with dehydrated strawberry, blueberry and red raspberry (5% w/w each) or 400 ppm ellagic acid for 3 weeks. Three berries with low (blueberry <100 ppm), moderate (strawberry - 500 ppm) and high (raspberry - 1500 ppm) ellagic acid content were chosen [17]. The ellagic acid dose was based on a similar short-term study in rats [32]. All berries were purchased as fresh produce locally (Lexington, KY) and processed as described elsewhere [44], in press). Ingredients for the diet were purchased individually from Dyets, Inc. (Bethlehem, PA). The control diet was slightly modified from the original composition for the AIN-93M diet [45] such that the carbohydrate calories were provided by corn starch and dextrose without the inclusion of sucrose (Table 2). The dried berries were added along with various ingredients at 5% (w/w) of the modified control diet and mixed in a Hobart mixer until homogenous. The corn starch component of the diet was adjusted in these diets such that all diets were approximately isocaloric after supplementation with berries. Ellagic acid was added at 400 ppm without any adjustment to the diet and mixed as described. Diets were stored at 4°C until use. Animals and the diets were weighed weekly to assess differences in diet intake and weight gain. They were euthanized by CO_2_-asphyxiation at the end of 3 weeks and liver was snap-frozen using liquid nitrogen in 2 aliquots, which were used for ^32^P-labeling and microarray analysis, respectively.

A modified version of the AIN-93M diet was fed to the mice as the control diet. The corn starch component was substituted for 5% berry diets such that the percentage of corn starch in these diets was 31.03%. No substitutions were done for the ellagic acid diet since this agent was added in insignificant quantities (0.04%). All diets were prepared as described in Materials and Methods. The diets were stored either at −80°C (>1 week) or 4°C (<1 week) until use. CHO- Carbohydrate. The composition of AIN-93M diet as described in [45].

### 4.4 Analysis of polar oxidative DNA adducts by ^32^P-postlabeling/TLC

DNA from liver was isolated as described [43] and 14 μg was digested to 3′-monophosphates using micrococcal nuclease/spleen phospodiesterase (Enzyme: DNA – 1:5, 5h, 37°C). After removing 2 μg of digest for normal nucleotide analysis, 10 μg digest was enriched for novel oxidative adducts by treatment with nuclease P1 (E: S-1:2.5, 1h, 37°C). Remaining 2 μg of the digest was enriched for 8-oxodG by PEI-cellulose TLC and 0.5–1 μg was labeled as described [10]. The 5′-^32^P-labeling of both enriched DNA adducts and normal nucleotides were done in parallel by T4-polynucleotide kinase and molar excess of [γ-^32^P] ATP as described earlier [12, 43]. Labeled adducts were separated by 2-D PEI-cellulose TLC using 50 mM sodium phosphate, pH 6.0 and 1 M formic acid in the D1 direction. D2 was perpendicular to D1 using a solvent mixture of isopropanol: 4M ammonium hydroxide: 8M urea in the ratio 3.3:1:1.6. Adducts with decreasing polarities in tissue DNA were eluted by increasing the sodium phosphate concentrations (50 mM – 1,000 mM) in the presence of 1 M formic acid (D1) but maintaining the same D2 solvent [12]. Adducts and normal nucleotides were visualized using Packard InstantImager and were counted individually. The enriched 8-oxodGp was labeled in parallel and chromatographed as described [10]. Adduct levels were calculated as relative adduct labeling (RAL) = (CPM adducts/CPM normal nucleotides) × 1/dilution factor and are expressed as adducts per10^6^ nucleotides (*in vitro* adducts) or per 10^9^ nucleotides (*in vivo* adducts).

### 4.5 Gene expression analysis

The liver tissue from 6 mice/group were pooled and total RNA was isolated using the phenol: chloroform extraction followed by DNase treatment. cDNA probes were synthesized from poly A^+^ RNA, using [α-^32^P]-ATP (3000 Ci/mmol). These probes were then hybridized to Atlas™ nylon mouse stress array (Clontech, Mountain View, CA; production discontinued) overnight in duplicate for each group. Following hybridization the membrane was exposed to X-ray film. The autoradiographic images were scanned with a MicroTek ScanMakerIII flat-bed scanner and then subjected to densitometric analysis using ArrayExplorer© to extract the gene intensities [46].

### 4.6 Statistics

A Scheffe’s t-test was used for comparing dose response studies. In vivo adduct levels were compared using non-parametric analysis (Kruskall-Wallis test). All statistical analyses, except those for the microarray data, were done using Graphpad Prism® software. A p-value <0.05 was considered to be statistically significant. The results are expressed as mean ± SE. For the microarray analyses, the data were normalized, using linear regression analysis and gene expression profiles were estimated as log2 of the ratio of the gene intensities of the control diet versus supplemented diet. The genes with significant (p<0.05) down- or up-regulation were identified.

## Figures and Tables

**Table 1.** Modulation of hepatic unidentified polar DNA adducts by diets supplemented with berries (5%w/w) or ellagic acid (400 ppm) in female CD-1 mice.

Adduct subgroup	P-1	P-2	PL-1	L-1	Total
	Adducts/ 10^9^ nucleotides (mean ± SE)				
**Control***(n*=*4)*	4810 ± 596	3398 ± 316	197 ± 32	3074 ± 697	11,479 ± 1128
**Strawberry***(n*=*4)*	3726 ± 1053	2182 ± 647	187 ± 23	2404 ± 502	8452 ± 1561
**Blueberry***(n*=*5)*	4982 ± 513	2243 ± 285	186 ± 37	1870 ± 248	8833 ± 668
**Red raspberry***(n*=*5)*	1803 ± 239 *	1090 ± 149 **	84 ± 16	1790 ± 433	4733 ± 333**
*p value*	*p < 0.05*	*p < 0.001*	*NS*	*NS*	*p < 0.001*
**Ellagic acid***(n*=*5)*	2060 ± 306 *	1207 ± 106 *	101 ± 8	2540 ± 254	5908 ± 540 *
*p value*	*p < 0.05*	*p < 0.01*	*NS*	*NS*	*p < 0.01*

**Table 2.** Comparison of AIN-93M diet composition to diets used in this study.

	Percent of composition			
Ingredients	AIN-93M diet	Control diet	Berry diet	Ellagic acid diet
Corn starch	46.6%	36.03%	31.03%	36.03%
Dextrose	15.5%	36.04%	36.04%	36.04%
Sucrose	10%	0%	0%	0%
Berries	–	0%	5%	0%
***Total CHO calories***	***72.1%***	***72.07%***	***72.07%***	***72.07%***
Casien	14%			
Soy Bean Oil	4%			
Fiber	5%			
AIN-93 Vitamin Mix	1%			
AIN-93 Mineral mix	3.5%			
L- Cysteine	0.18%			
Choline Bitartrate	0.25%			
